# Supplementary material for: Identification and Pharmacological Characterization of a Low-Liability Antinociceptive Bifunctional MOR/DOR Cyclic Peptide
Source: Molecules. 2023 Nov 11;28(22):7548. doi: 10.3390/molecules28227548 (PMC10674865; doi:10.3390/molecules28227548)
Supplement: Supplementary file 1 [file molecules-28-07548-s001.zip › molecules-2689107-supplementary.pdf]

Supplementary Materials

Identification and Pharmacological Characterization of a Low-Liability Antinociceptive Bifunctional MOR/DOR Cyclic Peptide

Yangmei Li [1,*], Shainnel O. Eans [2], Michelle Ganno-Sherwood [3], Abbe Eliasof [1], Richard A. Houghten [3] and Jay P. McLaughlin [2,*]

Table of Contents

1. NMR data
2. Eurofins DiscoverX SAFETYscan E/IC50 ELECT-78 assays

1. NMR data

Tyr-[D-Lys-Dap(Ant)-Thr-Gly] (CycloAnt). The cyclic peptide was obtained as a TFA salt after HPLC purification.

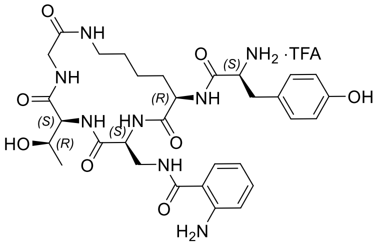

^{1}H NMR (400 MHz, DMSO-d_6) δ 0.99-1.22 (m, 2H), 1.03 (d, 3H, J = 6 Hz), 1.08-1.18 (m, 1H), 1.29-1.39 (m, 3H), 1.49-1.56 (m, 1H), 2.85-2.87 (m, 1H), 2.90 (m, 1H), 2.96-2.98 (m, 1H), 3.08-3.12 (m, 1H), 3.50 (td, 1H, J = 14, 6 Hz), 3.88 (d, 1H, J = 6.8 Hz), 3.90-3.94 (m, 1H), 3.99-4.02 (m, 2H), 4.08 (t, 1H, J = 6 Hz), 4.2-4.24 (m, 1H), 5.07 (d, 1H, J = 4.8 Hz), 6.34 (br.s, 2H), 6.49-6.52 (dd, 1H, J = 7.2, 1 Hz), 6.54 (d, 1H, J = 2 Hz), 6,72 (d, 3H, J = 8 Hz), 7.03 (d, 2 H, J = 8 Hz), 7.16 (td, 1H , J = 7.6, 1.6 Hz), 7.33 (t, 1H, J = 5.2 Hz), 7.46 (dd, 1H, J = 8, 1.2 Hz), 7.64 (d, 1H, J = 5.2 Hz), 7.72 (br.s, 1H), 8.13 (br.s, 2H), 8.29 (t, 1H, J = 5.6 Hz), 8.38 (t, 1H, J = 5.6 Hz), 8.43 (d, 1H, J = 7.2 Hz), 8.47 (d, 1H, J = 6.4 Hz), 9.39 (s, 1H)

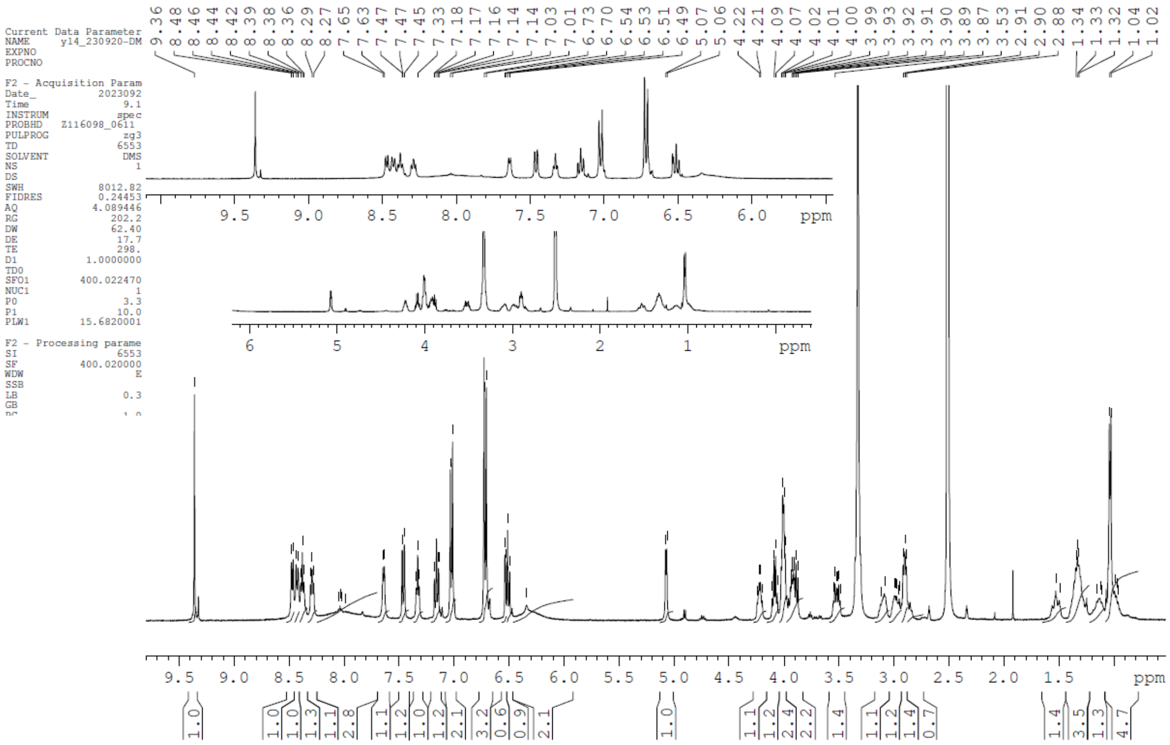

^{13}C NMR (100 MHz, DMSO-d_6) δ 20.1, 21.2, 28.1, 31.5, 36.8, 38.2, 40.7, 43.8, 53.6, 54.2, 56.4, 60.2, 66.3, 114.9, 115.3, 115.7, 116.9, 118.7, 125.2, 128.8, 130.9, 132.5, 149.7, 157.1, 158.4, 158.7, 168.2, 169.2, 170.5, 170.6, 172.1

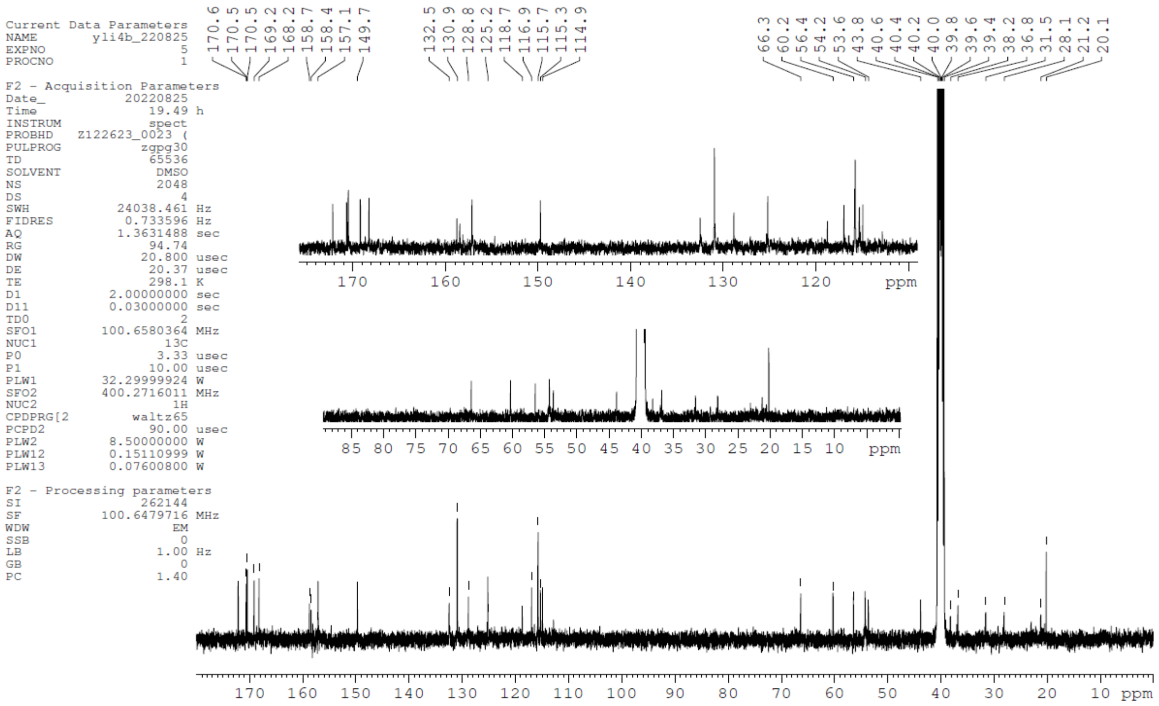

DEPT135

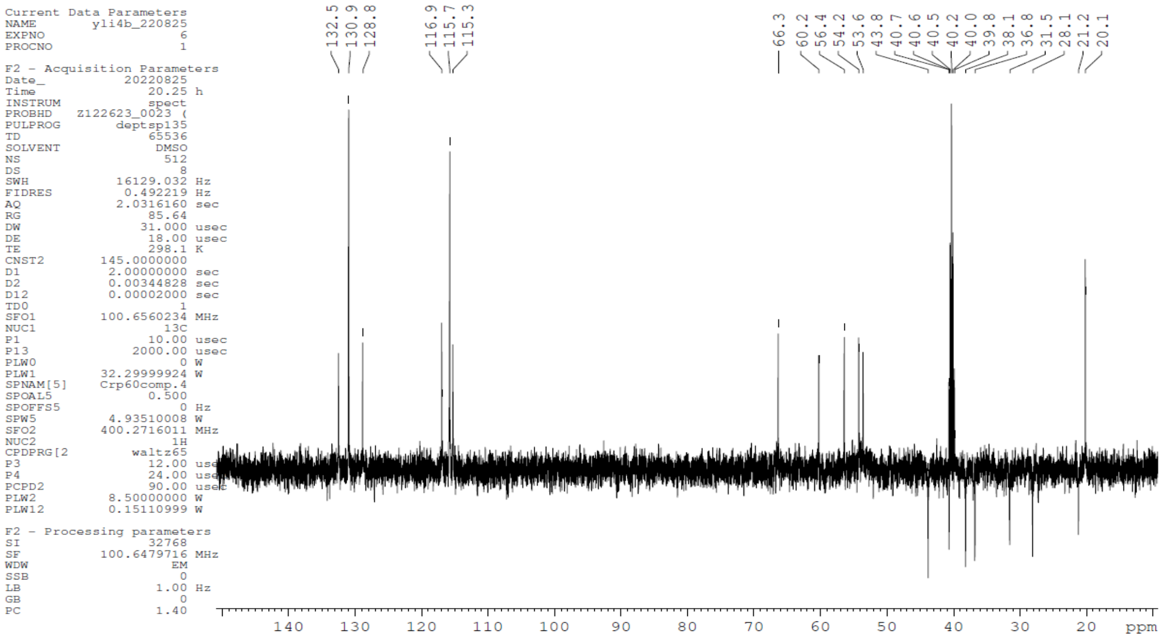

F NMR: (376 MHz): d -73.7 (s)

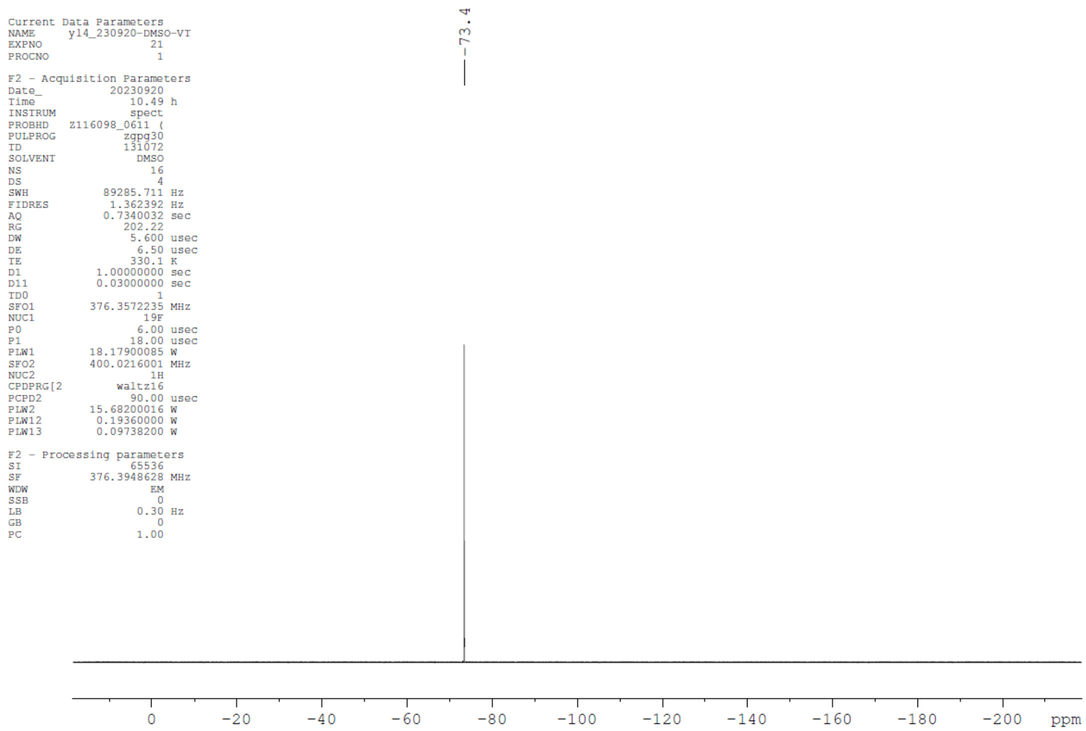

TOCSY

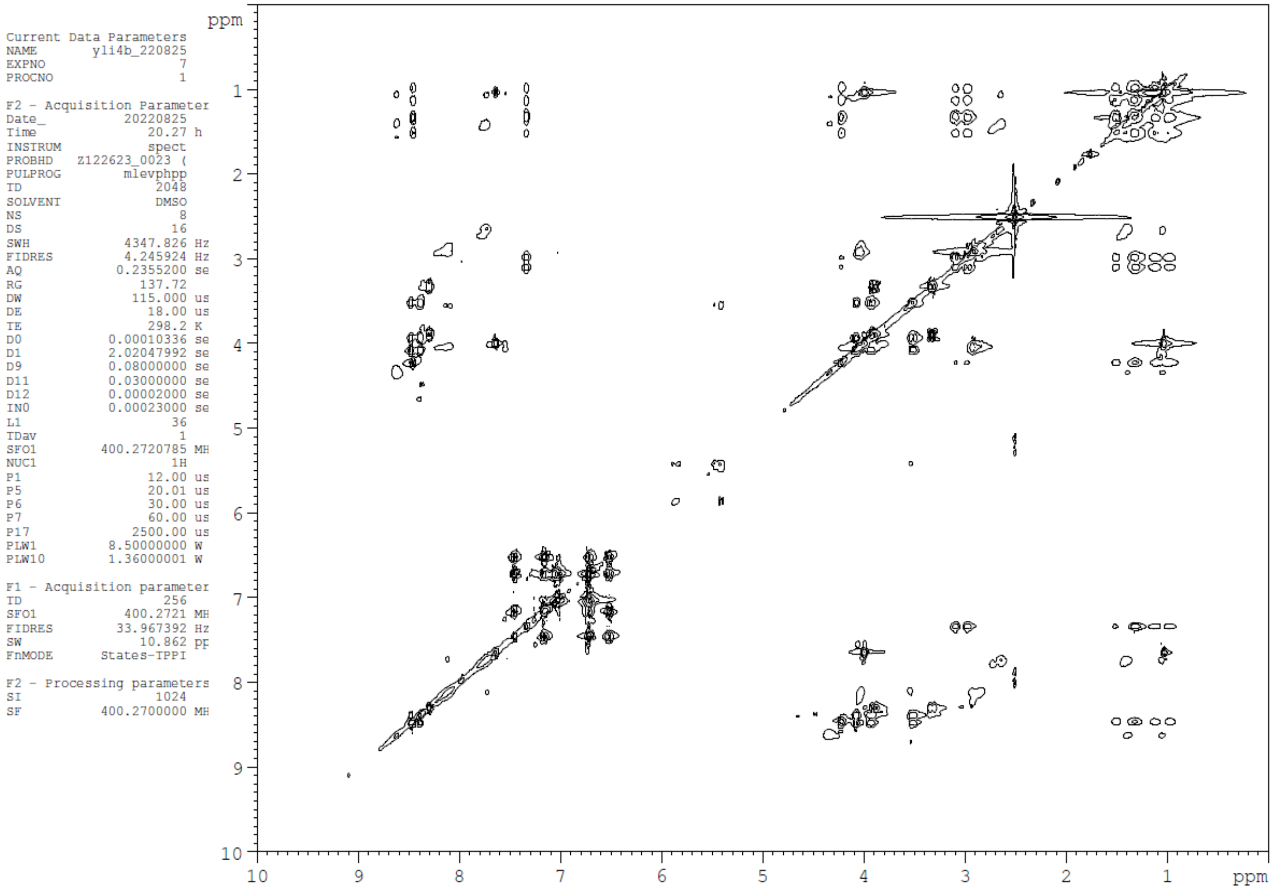
Current Data Parameters
NAME yli4b_220825
EXPNO 7
PROCNO 1
F2 - Acquisition Parameter
Date_ 20220825
Time 20.27 h
INSTRUM spect
PROBHD Z122623_0023 (
PULPROG mlevphpp
TD 2048
SOLVENT DMSO
NS 8
DS 16
SWH 4347.826 Hz
FIDRES 4.245924 Hz
AQ 0.2355200 se
RG 137.72
DW 115.000 us
DE 18.00 us
TE 298.2 K
D0 0.00010336 se
D1 2.02047992 se
D9 0.08000000 se
D11 0.03000000 se
D12 0.00002000 se
IN0 0.00023000 se
L1 36
TDav 1
SFO1 400.2720785 MH
NUC1 1H
P1 12.00 us
P5 20.01 us
P6 30.00 us
P7 60.00 us
P17 2500.00 us
PLW1 8.50000000 W
PLW10 1.36000001 W
F1 - Acquisition parameter
TD 256
SFO1 400.2721 MH
FIDRES 33.967392 Hz
SW 10.862 pp
FnMODE States-TPPI
F2 - Processing parameters
SI 1024
SF 400.2700000 MH
ppm
1
2
3
4
5
6
7
8
9
10
10
9
8
7
6
5
4
3
2
1
ppm

Leu-[D-Lys-Dap(Ant)-Thr-Gly] (CycloAnt-Leu). The cyclic peptide was obtained as a TFA salt after HPLC purification.

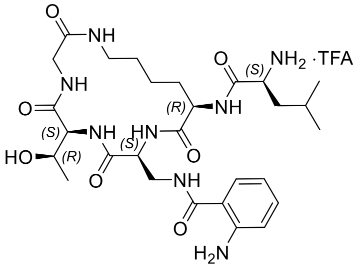

^{1}H NMR (400 MHz, DMSO-d_6) δ 8.83 (d, 1H, *J* = 7.2), 8.57 (d, 1H, *J* = 6.0 Hz), 8.38 (t, 1H, *J* = 6.0 Hz), 8.26 (t, 1H, *J* = 6.0 Hz), 8.15 (s, 3H), 7.66 (d, 1H, *J* = 6.0 Hz), 7.48 (dd, 1H, *J* = 8.28 Hz, 1.2 Hz), 7.41 (t, 1H, *J* = 5.6 Hz), 7.16 (td, 1H, *J* = 7.68 Hz, 1.2 Hz), 6.73 (dd, 1H, J = 8.2 Hz, 0.68 Hz), 6.51 (t, 1H, *J* = 7.6 Hz), 4.19 (q, 1H, *J* =), 4.12 (q, 1H, *J* = 6.0 Hz), 4.03 (d, 1H, *J* = 5.96 Hz), 4.03 (m, 1H), 3.98 (m, 1H), 3.91 (m, 1H), 3.88 (dd, 1H, *J* = 16.4 Hz, 6.8 Hz), 3.53 (dt, 1H, *J* = 14.0 Hz, 5.6 Hz), 3.36 (dd, 1H, *J* = 16.0 Hz, 4.8 Hz), 3.18 – 3.10 (m, 1H), 3.01 – 2.94 (m, 1H), 1.61 (m, 5H), 1.46 – 1.21 (m, 4H), 1.03 (d, 2H, *J* = 6.0 Hz), 0.92 (d, 6H, *J* = 5.6 Hz)

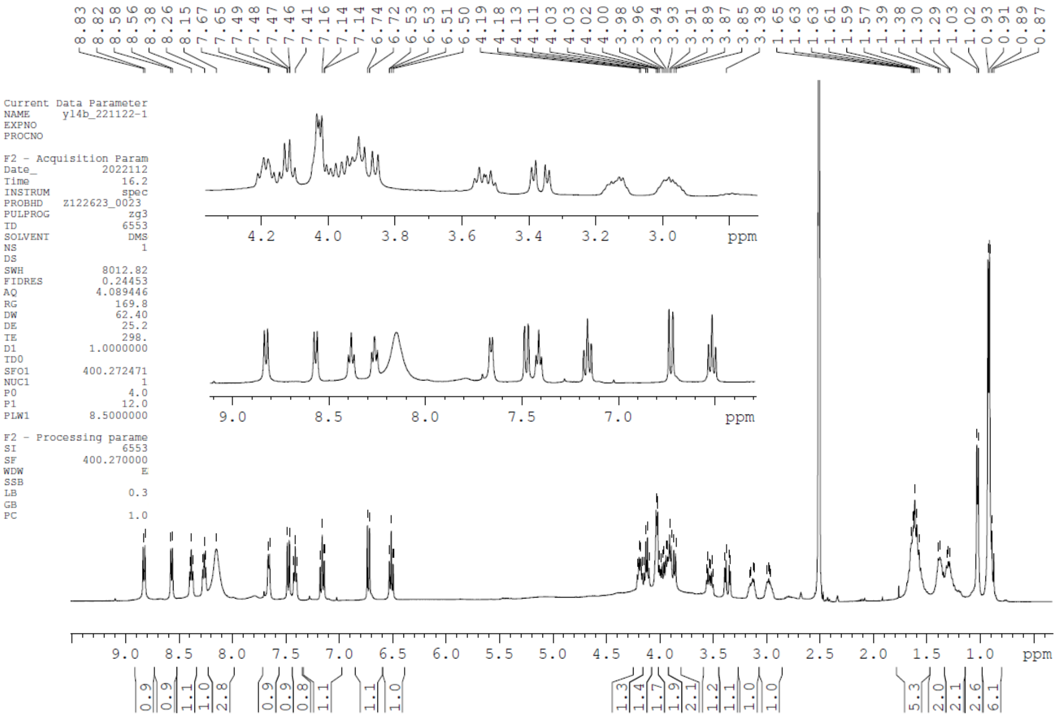

^{13}C NMR (400 MHz, DMSO-d_6) δ 172.36, 170.72, 170.51, 170.48, 169.53, 169.151, 158.75, 158.43, 149.83, 132.50, 128.85, 118.78, 116.94, 115.81, 115.22, 114.72, 66.32, 60.03, 56.44, 54.41, 51.39, 43.83, 40.55, 40.39, 38.17, 31.62, 28.23, 24.08, 23.22, 22.09, 21.71, 20.11

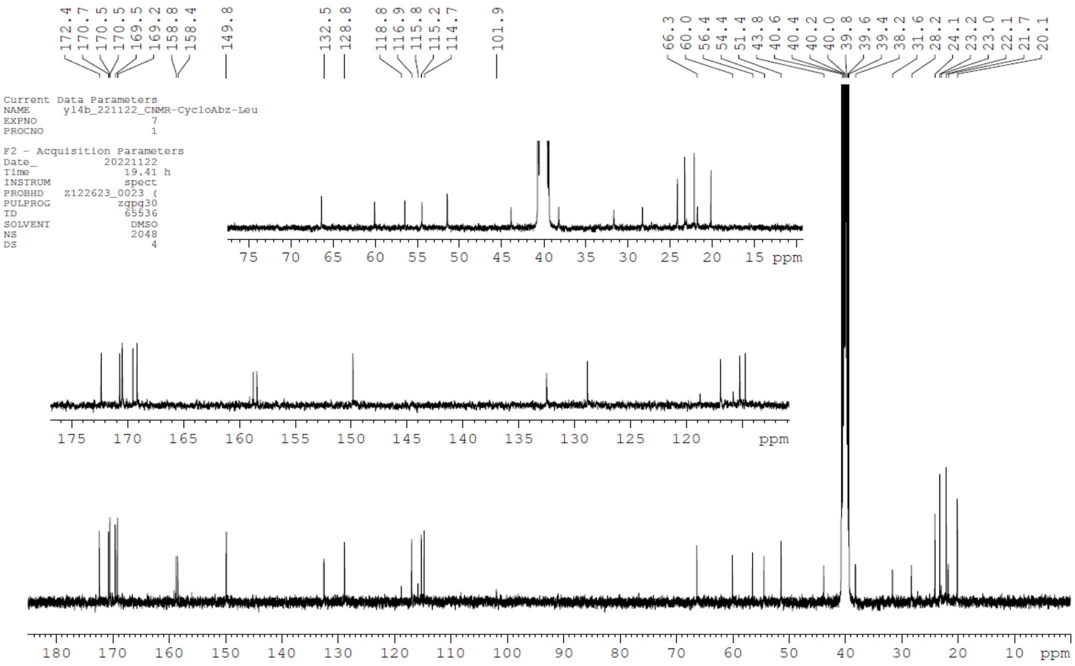

DEPT135

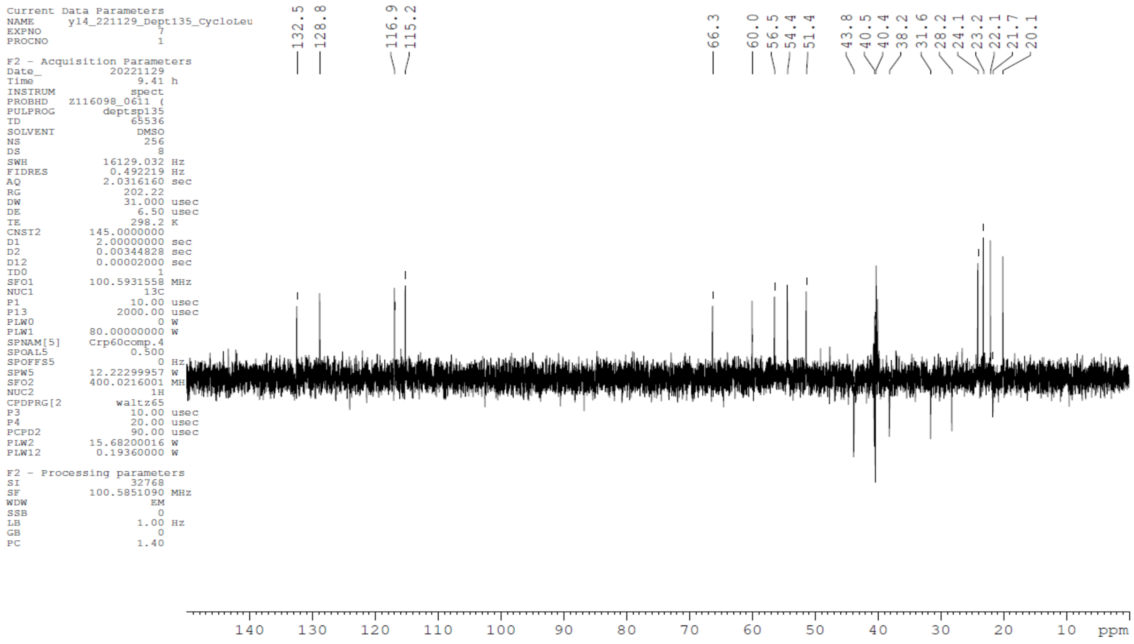

TOCSY

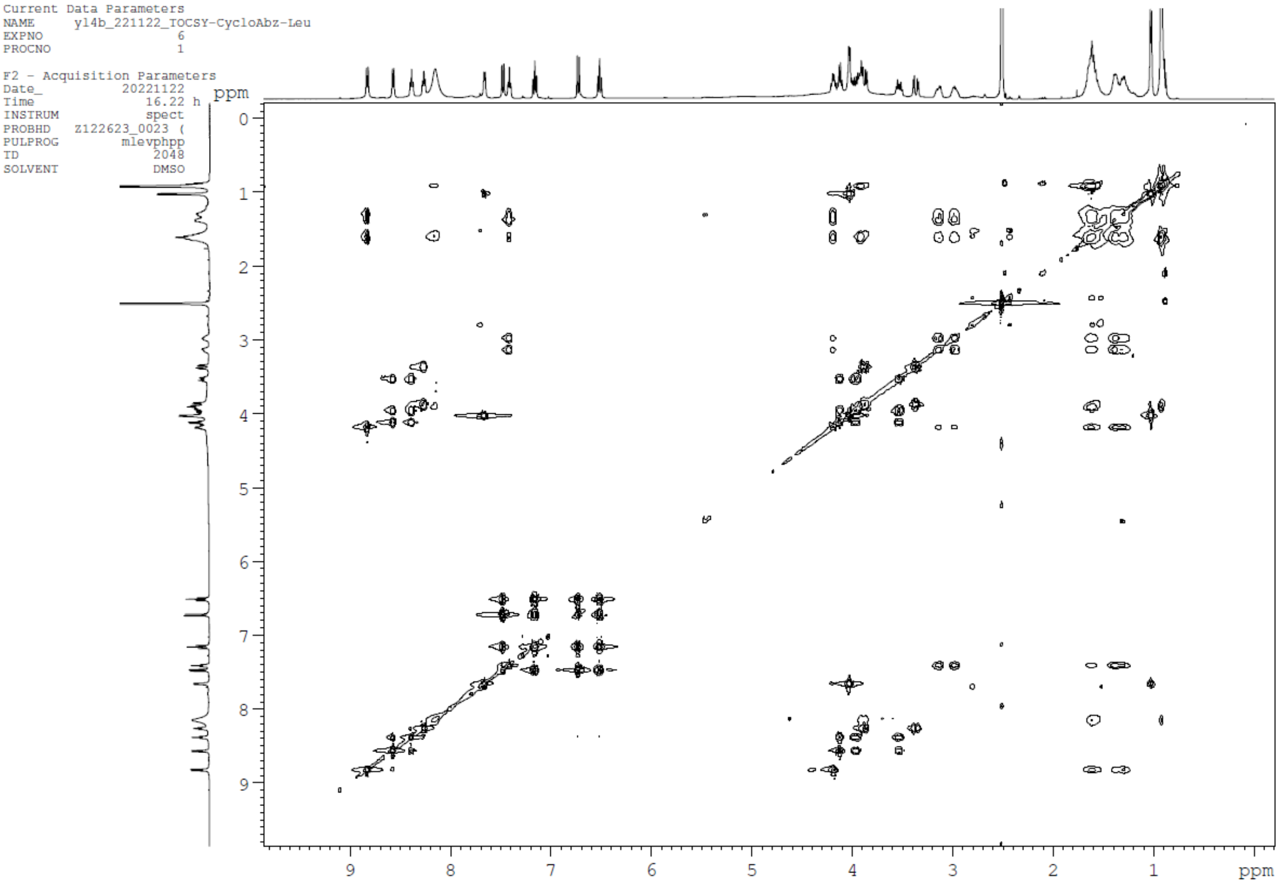
Current Data Parameters
NAME y14b_221122_TOCSY-CycloAbz-Leu
EXPNO 6
PROCNO 1
F2 - Acquisition Parameters
Date_ 20221122
Time 16.22 h
INSTRUM spect
PROBHD Z122623_0023 (
PULPROG mlevphpp
TD 2048
SOLVENT DMSO
ppm

2. Safety47 Panel Dose Response

Results: Summary Table

Compound Name	Order ID	Target Class	Assay Name	Mode	Assay Target	Result Type	Value Prefix	RC50 (μM)	Hill	Curve Bottom	Curve Top	Max Response
USCYL05	US073-0023285-O	GPCR	Calcium Flux	Agonist	ADORA2A	EC50	>	5				0
USCYL05	US073-0023285-O	GPCR	Calcium Flux	Agonist	ADRA1A	EC50	>	5				2.7
USCYL05	US073-0023285-O	GPCR	Calcium Flux	Agonist	AVPR1A	EC50	>	5				1.19
USCYL05	US073-0023285-O	GPCR	Calcium Flux	Agonist	CCKAR	EC50	>	5				0
USCYL05	US073-0023285-O	GPCR	Calcium Flux	Agonist	CHRM1	EC50	>	5				1.1
USCYL05	US073-0023285-O	GPCR	Calcium Flux	Agonist	CHRM3	EC50	>	5				0
USCYL05	US073-0023285-O	GPCR	Calcium Flux	Agonist	EDNRA	EC50	>	5				0
USCYL05	US073-0023285-O	GPCR	Calcium Flux	Agonist	HRH1	EC50	>	5				1.47
USCYL05	US073-0023285-O	GPCR	Calcium Flux	Agonist	HTR2A	EC50	>	5				0
USCYL05	US073-0023285-O	GPCR	Calcium Flux	Agonist	HTR2B	EC50	>	5				0
USCYL05	US073-0023285-O	GPCR	Calcium Flux	Antagonist	ADORA2A	IC50	>	5				10.1
USCYL05	US073-0023285-O	GPCR	Calcium Flux	Antagonist	ADRA1A	IC50	>	5				5.86
USCYL05	US073-0023285-O	GPCR	Calcium Flux	Antagonist	AVPR1A	IC50	>	5				0
USCYL05	US073-0023285-O	GPCR	Calcium Flux	Antagonist	CCKAR	IC50	>	5				0
USCYL05	US073-0023285-O	GPCR	Calcium Flux	Antagonist	CHRM1	IC50	>	5				0
USCYL05	US073-0023285-O	GPCR	Calcium Flux	Antagonist	CHRM3	IC50	>	5				2.03
USCYL05	US073-0023285-O	GPCR	Calcium Flux	Antagonist	EDNRA	IC50	>	5				2.26
USCYL05	US073-0023285-O	GPCR	Calcium Flux	Antagonist	HRH1	IC50	>	5				8.01
USCYL05	US073-0023285-O	GPCR	Calcium Flux	Antagonist	HTR2A	IC50	>	5				0
USCYL05	US073-0023285-O	GPCR	Calcium Flux	Antagonist	HTR2B	IC50	>	5				2.57
USCYL05	US073-0023285-O	GPCR	cAMP	Agonist	ADRA2A	EC50	>	5				0
USCYL05	US073-0023285-O	GPCR	cAMP	Agonist	ADRB1	EC50	>	5				0
USCYL05	US073-0023285-O	GPCR	cAMP	Agonist	ADRB2	EC50	>	5				0
USCYL05	US073-0023285-O	GPCR	cAMP	Agonist	CHRM2	EC50	>	5				13.68
USCYL05	US073-0023285-O	GPCR	cAMP	Agonist	CNR1	EC50	>	5				6.81
USCYL05	US073-0023285-O	GPCR	cAMP	Agonist	CNR2	EC50	>	5				10.78

eurofins | DISCOVERY

Results: Summary Table (cont.)

Compound Name	Order ID	Target Class	Assay Name	Mode	Assay Target	Result Type	Value Prefix	RC50 (μM)	Hill	Curve Bottom	Curve Top	Max Response
USCYL05	US073-0023285-O	GPCR	cAMP	Agonist	DRD1	EC50	>	5				0.11
USCYL05	US073-0023285-O	GPCR	cAMP	Agonist	DRD2S	EC50	>	5				0
USCYL05	US073-0023285-O	GPCR	cAMP	Agonist	HRH2	EC50	>	5				0
USCYL05	US073-0023285-O	GPCR	cAMP	Agonist	HTR1A	EC50	>	5				1.37
USCYL05	US073-0023285-O	GPCR	cAMP	Agonist	HTR1B	EC50	>	5				6.52
USCYL05	US073-0023285-O	GPCR	cAMP	Agonist	OPRD1	EC50	=	0.00336	1.21	0	100	100.82
USCYL05	US073-0023285-O	GPCR	cAMP	Agonist	OPRK1	EC50	>	5				0.78
USCYL05	US073-0023285-O	GPCR	cAMP	Agonist	OPRM1	EC50	=	0.01404	1.48	0	100	98.78
USCYL05	US073-0023285-O	GPCR	cAMP	Antagonist	ADRA2A	IC50	>	5				2.69
USCYL05	US073-0023285-O	GPCR	cAMP	Antagonist	ADRB1	IC50	>	5				0
USCYL05	US073-0023285-O	GPCR	cAMP	Antagonist	ADRB2	IC50	>	5				0
USCYL05	US073-0023285-O	GPCR	cAMP	Antagonist	CHRM2	IC50	>	5				6.71
USCYL05	US073-0023285-O	GPCR	cAMP	Antagonist	CNR1	IC50	>	5				6.13
USCYL05	US073-0023285-O	GPCR	cAMP	Antagonist	CNR2	IC50	>	5				0
USCYL05	US073-0023285-O	GPCR	cAMP	Antagonist	DRD1	IC50	>	5				0
USCYL05	US073-0023285-O	GPCR	cAMP	Antagonist	DRD2S	IC50	>	5				2.83
USCYL05	US073-0023285-O	GPCR	cAMP	Antagonist	HRH2	IC50	>	5				0
USCYL05	US073-0023285-O	GPCR	cAMP	Antagonist	HTR1A	IC50	>	5				4.16
USCYL05	US073-0023285-O	GPCR	cAMP	Antagonist	HTR1B	IC50	>	5				0.52
USCYL05	US073-0023285-O	GPCR	cAMP	Antagonist	OPRD1	IC50	>	5				0
USCYL05	US073-0023285-O	GPCR	cAMP	Antagonist	OPRK1	IC50	>	5				1.38
USCYL05	US073-0023285-O	GPCR	cAMP	Antagonist	OPRM1	IC50	>	5				0
USCYL05	US073-0023285-O	Ion Channel	Ion Channel	Blocker	CAV1.2	IC50	>	5				18.67
USCYL05	US073-0023285-O	Ion Channel	Ion Channel	Blocker	GABAA	IC50	>	5				25.09
USCYL05	US073-0023285-O	Ion Channel	Ion Channel	Blocker	hERG	IC50	>	5				4.3
USCYL05	US073-0023285-O	Ion Channel	Ion Channel	Blocker	HTR3A	IC50	>	5				18.94

Results: Summary Table (cont.)

Compound Name	Order ID	Target Class	Assay Name	Mode	Assay Target	Result Type	Value Prefix	RC50 (µM)	Hill	Curve Bottom	Curve Top	Max Response
USCYL05	US073-0023285-0	Ion Channel	Ion Channel	Blocker	KvLQT1/minK	IC50	>	5				7.33
USCYL05	US073-0023285-0	Ion Channel	Ion Channel	Blocker	nAChR(a4/b2)	IC50	>	5				0
USCYL05	US073-0023285-0	Ion Channel	Ion Channel	Blocker	NAV1.5	IC50	>	5				0
USCYL05	US073-0023285-0	Ion Channel	Ion Channel	Blocker	NMDAR (1A/2B)	IC50	>	5				0
USCYL05	US073-0023285-0	Ion Channel	Ion Channel	Opener	GABAA	EC50	>	5				1.74
USCYL05	US073-0023285-0	Ion Channel	Ion Channel	Opener	HTR3A	EC50	>	5				9.8
USCYL05	US073-0023285-0	Ion Channel	Ion Channel	Opener	KvLQT1/minK	EC50	>	5				0.46
USCYL05	US073-0023285-0	Ion Channel	Ion Channel	Opener	nAChR(a4/b2)	EC50	>	5				0
USCYL05	US073-0023285-0	Ion Channel	Ion Channel	Opener	NMDAR (1A/2B)	EC50	>	5				0
USCYL05	US073-0023285-0	Kinases	Binding	Inhibitor	INSR	IC50	>	5				0
USCYL05	US073-0023285-0	Kinases	Binding	Inhibitor	LCK	IC50	>	5				0
USCYL05	US073-0023285-0	Kinases	Binding	Inhibitor	ROCK1	IC50	>	5				7.68
USCYL05	US073-0023285-0	Kinases	Binding	Inhibitor	VEGFR2	IC50	>	5				0
USCYL05	US073-0023285-0	NHR	NHR Nuclear Translocation	Agonist	AR	EC50	>	5				0
USCYL05	US073-0023285-0	NHR	NHR Nuclear Translocation	Antagonist	AR	IC50	>	5				1.11
USCYL05	US073-0023285-0	NHR	NHR Protein Interaction	Agonist	GR	EC50	>	5				0
USCYL05	US073-0023285-0	NHR	NHR Protein Interaction	Antagonist	GR	IC50	>	5				5.74
USCYL05	US073-0023285-0	Non-Kinase Enzymes	Enzymatic	Inhibitor	AChE	IC50	>	5				0
USCYL05	US073-0023285-0	Non-Kinase Enzymes	Enzymatic	Inhibitor	COX1	IC50	>	5				0
USCYL05	US073-0023285-0	Non-Kinase Enzymes	Enzymatic	Inhibitor	COX2	IC50	>	5				0
USCYL05	US073-0023285-0	Non-Kinase Enzymes	Enzymatic	Inhibitor	MAOA	IC50	>	5				0
USCYL05	US073-0023285-0	Non-Kinase Enzymes	Enzymatic	Inhibitor	PDE3A	IC50	>	5				0.74
USCYL05	US073-0023285-0	Non-Kinase Enzymes	Enzymatic	Inhibitor	PDE4D2	IC50	>	5				0
USCYL05	US073-0023285-0	Transporter	Transporter	Blocker	DAT	IC50	>	5				12.09
USCYL05	US073-0023285-0	Transporter	Transporter	Blocker	NET	IC50	>	5				15.42
USCYL05	US073-0023285-0	Transporter	Transporter	Blocker	SERT	IC50	>	5				3.65
